# Hypertension Management in Women With a Multidisciplinary Approach

**DOI:** 10.1016/j.mayocp.2024.10.005

**Published:** 2024-12-29

**Authors:** Niloofar Nobakht, Yalda Afshar, Marmar Vaseghi, Zhaoping Li, Ines Donangelo, Helen Lavretsky, Thalia Mok, Christina S. Han, Susanne B. Nicholas

**Affiliations:** Division of Nephrology, Department of Medicine at the David Geffen School of Medicine at University of California, Los Angeles, CA, USA.; Division of Maternal Fetal Medicine, Department of Obstetrics and Gynecology, at the David Geffen School of Medicine at University of California, Los Angeles, CA, USA.; Division of Cardiology, Department of Medicine, at the David Geffen School of Medicine at University of California, Los Angeles, CA, USA.; Division of Clinical Nutrition, Department of Medicine, at the David Geffen School of Medicine at University of California, Los Angeles, CA, USA.; Division of Endocrinology, Department of Medicine, at the David Geffen School of Medicine at University of California, Los Angeles, CA, USA.; Department of Psychiatry at the David Geffen School of Medicine at University of California, Los Angeles, CA, USA.; Division of Maternal Fetal Medicine, Department of Obstetrics and Gynecology, at the David Geffen School of Medicine at University of California, Los Angeles, CA, USA.; Division of Maternal Fetal Medicine, Department of Obstetrics and Gynecology, at the David Geffen School of Medicine at University of California, Los Angeles, CA, USA.; Division of Nephrology, Department of Medicine, at the David Geffen School of Medicine at University of California, Los Angeles, CA, USA.

## Abstract

Current clinical practice guidelines were established by several organizations to guide the diagnosis and treatment of hypertension in men and women in a similar manner despite data demonstrating differences in underlying mechanisms. Few publications have provided a contemporary and comprehensive review focused on characteristics of hypertension that are unique to women across their life spectrum. We performed a computerized search using PubMed, OVID, EMBASE, and Cochrane library databases between 1995 and 2023 that highlighted relevant clinical studies, challenges to the management of hypertension in women, and multidisciplinary approaches to hypertension control in women, including issues unique to racial and ethnic minority groups. Despite our current understanding of underlying mechanisms and strategies to manage hypertension in women, numerous challenges remain. Here, we discuss potential factors contributing to hypertension in women, differences related to effects of lifestyle modifications and drug therapy between men and women, the impact of sleep, and the importance of recognizing disparities in socioeconomic conditions and access to care. This review outlines several opportunities for future studies to fill gaps in knowledge to achieve optimal control of hypertension in women using a multidisciplinary approach, particularly related to sex-specific treatment approaches while considering socioeconomic conditions and life stages from premenopause through the transition to menopause.

Hypertension is a significant global health problem and an important cause of morbidity and mortality worldwide, serving as an important risk factor for cardiovascular disease (CVD), chronic kidney disease (CKD), stroke, and dementia. The prevalence of hypertension was estimated to be 1.27 billion globally, with ~626 million women living with hypertension, worldwide.^1^ Nearly 1 of 2 adults in the United States has hypertension (116 million), with more than 50% reported to be women (44.9 million women and 40.8 million men).^2^ Optimal management of hypertension can improve both cardiovascular outcomes and mortality. It has become evident that, although hypertension is more prevalent in men,^3^ the risk factors for developing hypertension and the blood pressure thresholds for its diagnosis may differ by sex. However, the current guideline does not provide different blood pressure values for men and women.^4^ Specific risk factors related to lifecycle changes are present in women that affect both development and management of hypertension. A multidisciplinary approach may lead to better blood pressure (BP) control in hypertensive females. This review, which was conducted using PubMed, OVID, EMBASE, and Cochrane library databases between 1995 and 2023, provides a comprehensive summary of the presentation and treatment of hypertension as it relates to women across the lifespan and includes reference to specific racial and ethnic minority groups. It describes relevant clinical studies and ongoing challenges and outlines unanswered questions pertinent to the optimal management of hypertension in women.

The 2017 American College of Cardiology/American Heart Association Clinical Practice hypertension guideline recognizes the importance of risk calculators to determine when to initiate antihypertensive therapy. It also includes major changes in BP values, lowering the treatment target from less than 140/90 mm Hg to less than 130/80 mm Hg,^5^ and supporting improved cardiovascular outcomes with treatment aimed at lower targets. These changes were derived from evidence of increased CVD risk associated with BP 130–139/80–89 mm Hg, along with data supporting better associated cardiovascular outcomes.^3^ According to the new guideline, BP is “normal” if it is less than 120/80 mm Hg. The guideline also categorizes hypertension by stages: patients with systolic BP between 130 and 139 mm Hg or diastolic BP between 80 and 89 mm Hg have stage 1 hypertension, whereas those with a systolic BP greater than or equal to 140 mm Hg or diastolic BP greater than or equal to 90 mm Hg have stage 2 hypertension.^5,6^ The guideline remains consistent for both men and women, targeting BP less than 130/80 mm Hg for patients with coexisting coronary artery disease (CAD), diabetes mellitus (DM), CKD, peripheral vascular disease, and cerebral vascular accident (CVA) events without consideration for patient sex or physiological state at different life cycles.^5,6^ In this review, several sex-specific strategies to achieve optimal management of hypertension in women using a multidisciplinary approach are discussed. Importantly, socioeconomic conditions and transitions across different life stages from premenopause to menopause are also considered.

## RISK OF HYPERTENSION ACROSS THE WOMAN’S LIFESPAN

The prevalence of hypertension increases with age in both sexes, particularly after 40 years of age.^7^ In women, the prevalence is slightly lower than in men between the ages of 40 to 59 years (59.4% in men vs 49.9% in women). However, by 60 years of age, women have nearly caught up with men, with hypertension prevalence of 75.2% in men and 73.9% in women.^7^

The current guideline does not provide specific recommendations related to the management of hypertension by sex. According to all major society guidelines, accurate measurements, diagnosis of hypertension with repeat visits, ambulatory blood pressure monitoring (ABPM), or home BP monitoring via commercial devices are important in the diagnosis of hypertension for both women and men. Home and ABPM measurements are less likely to be confounded by white coat or masked hypertension. Although nearly all major clinical trials use clinic BP readings, ABPM can supplement these readings because data indicate that ABPM is a stronger predictor of clinical outcomes compared with clinic measurements^8–10^ and allows for measurement of nocturnal pressures.^11–13^

A 10% to 20% decrease in nighttime BP compared with daytime is expected,^5,6^ and patients who are nighttime “nondippers” or “reverse dippers” have higher risk for stroke, cardiac hypertrophy, and silent CVA events.^8,11,14,15^ Reverse and nondipping BP, for women more than men, increase the risk of cardiovascular events with age. Middle-aged and postmenopausal women who are nondippers are at highest risk for cardiovascular events, particularly if they present with comorbid conditions.^16^ Nondipping is associated with a higher left ventricular mass in both sexes, although this is the case to a greater extent in women.^17^

Ambulatory BP monitoring has also allowed for assessment of important changes in BP over the lifetime of males vs females. Using ABPM data in 15,913 women and 14,600 men, diastolic BP was noted to fall at a very early age (22.3 years) in women, whereas in men, diastolic BP begins to fall at age 46.5 years.^18^ Finally, there is a greater prevalence of masked hypertension in men than women, as detected by ABPM.^19–21^

## HYPERTENSION DURING REPRODUCTIVE AGE

Epidemiological studies have shown associations between clinical BP or diagnosed hypertension in youth with atherosclerotic CVD and premature mortality.^22^ The menstrual cycle affects arterial compliance and may change systolic and diastolic BP; however, these variations in normotensive women appear to be modest and larger studies are needed.

Although most patients have primary (essential) hypertension, the proportion of individuals affected by secondary hypertension is higher in women of reproductive age, despite a lower prevalence of primary hypertension in this age group. The diagnosis of secondary causes requires a high index of suspicion, and appropriate testing based on clinical presentation. Early detection of secondary hypertension is pivotal because timely diagnosis and management of underlying condition allow for prevention of hypertension-mediated organ damage (HMOD).

Several recognized endocrine causes of secondary hypertension include primary aldosteronism (PA), pheochromocytoma, Cushing syndrome, thyroid disease, acromegaly, and hyperparathyroidism.^23^ Primary aldosteronism is the most common cause of secondary hypertension, with a prevalence of ~5% among patients with hypertension, and up to 20% in individuals with resistant hypertension.^24,25^ The most common cause of PA is bilateral adrenal hyperplasia, followed by aldosterone-producing adrenal adenoma, representing in 60% and 30% of cases, respectively.^26^ Unilateral PA is more common in men, whereas bilateral adrenal hyperplasia is more common in women.^27^ Observed differences in incidence are further modified by age, where women diagnosed with unilateral adrenal hyperplasia are more likely to be younger than men. The inverse of this is true for bilateral adrenal hyperplasia, as male patients with this diagnosis tend to be younger than female patients.^27^ The prevalence of PA is similar in men and women, but estrogen can affect plasma renin concentration (PRC), and may interfere with laboratory investigation of PA. Although there is good correlation between plasma renin activity (PRA) and PRC, estrogen use or preovulatory (luteal phase) estrogen surge in women is associated with false positive case detection testing when using PRC but not PRA. Plasma renin activity may be more suitable for PA case detection in premenopausal women or in those taking oral estrogen.^26,28^

Salt-sensitive BP characterized by an increase in BP following salt loading or a drop in BP following salt depletion is reported in up to 50% of hypertensive patients.^29^ This is more common in women than men and increases with age.^30,31^ The mineralocorticoid pathway may be implicated in the mechanism for salt-sensitive hypertension in younger women. Premenopausal women may have heightened aldosterone production to stimuli and expression of endothelial mineralocorticoid receptor (MR) mediated by sex hormones that lead to endothelial dysfunction and hypertension. Therefore, MR antagonism may be preferred in the treatment of salt-sensitive hypertension in premenopausal women.^32^ The role of MR activation and augmented aldosterone production diminishes after menopause. In postmenopausal women, as in men, dysfunctional renal physiology leading to impaired natriuresis is the main contributor to salt-sensitive hypertension.^16,32^

Renovascular hypertension is among the most common causes of secondary hypertension, primarily from atherosclerotic lesions (60%–90%) followed by fibromuscular dysplasia (10%–30%).^33^ Fibromuscular dysplasia has a female/male ratio of 84%/16% in the United States and is predominantly diagnosed in middle-aged subjects.^33^ It is a noninflammatory disease of medium-size arteries which may lead to stenosis, occlusion, and or dissection.^34^ Approximately 90% of these patients have hypertension, and a significant proportion of them may present with major vascular events such as CAD, cerebrovascular and renal events including ischemia, infarction, and renal failure. Earlier diagnosis and vascular interventional treatment with angioplasty will improve outcome and adverse events (Table 1).^33,34^

### Preconception Care

Optimizing the care of the pregnant person with hypertension, even before pregnancy, is the ideal. As such, a preconception consultation with a multidisciplinary care team, including maternal-fetal medicine, is recommended in reproductive-age people who desire pregnancy.^43^ The goal of preconception care is to affirm pregnancy intention, mitigate potential harm, and recognize modifiable risk factors related to pregnancy while stratifying pregnancies on a continuum of low to high risk.^43^ Although most patients with well-controlled hypertension will have uncomplicated pregnancies, patients with hypertension should have a clear understanding of the possible complications in pregnancy, potential effects of pregnancy on BP, and the need for heightened increased maternal and fetal surveillance. Patients with modifiable risk factors such as obesity, smoking, and poorly controlled diabetes may benefit from a discussion on lifestyle modifications. These include weight loss, diet, exercise, and smoking cessation to improve BP control and decrease rates of hypertension disorders of pregnancy (HDP) and associated adverse maternal and fetal outcomes.^44,45^ All patients in need of renin-angiotensin-aldosterone (RAAS) blockade agents should have a discussion of effective contraceptive options along with negative pregnancy test before initiation of therapy given teratogenicity of these agents including fetal cardiac and kidney defects.^46^

### Contraceptives

Drug-induced hypertension by estrogen and progestins is another cause of secondary hypertension among women. The first cases of severe BP elevations after oral contraceptive pills (OCPs) were noted in the 1960s.^47^ A few decades later, changes in the RAAS have been noted in users of OCPs, and cumulative use of OCPs has been found to be associated with a higher risk of hypertension.^48^ Combined hormonal contraceptives have remained a more popular option among users and newer formulations of OCPs containing natural estrogens and progestins with antimineralocorticoid effects (ie, drospirenone) to decrease or reverse the association between OCP use and BP elevations.^49^ Approximately 10% of reproductive-aged women have systolic BP greater than or equal to 140 mm Hg or diastolic BP greater than or equal to 90 mm Hg, and almost 20% have systolic BP greater than or equal to 130 mm Hg or diastolic BP greater than or equal to 80 mm Hg in the United States.^50^ Therefore, standardized practices to identify contraindications to OCPs are needed to ensure safety of use. Hormonal intrauterine devices which only contain progestin are generally considered safe for individuals with high BP.^51^ A preexisting understanding of the risks and benefits associated with hormonal contraceptive agents is recommended.

## HYPERTENSIVE DISORDERS OF PREGNANCY

Hypertensive disorders of pregnancy, including pre-eclampsia, eclampsia, and gestational hypertension affect ~10% of all pregnancies and are associated with increased morbidity and mortality for the mother and the fetus.^52–54^ Hypertensive disorders of pregnancy include both pregestation and gestational hypertension, with maternal and neonatal complications.^55,56^ According to the 2019 National Center for Health Statistics data, more than 83,000 pregnant women in the United States had chronic hypertension predating their pregnancies^57^ and ~46,000 maternal deaths were associated with pre-eclampsia or gestational hypertension. The prevalence of HDP is expected to continue to rise with the obesity epidemic and increasing maternal age.^58^ Data from a large cohort of 433,430 women in the United States confirmed an increase in maternal CAD, stroke, and mortality within 5 years post-delivery among women with HDP.^59^ It is also associated with peripheral vascular disease and CKD.^55^ Across all ethnicities, HDPs are associated with significantly increased risk for developing CKD and chronic hypertension^60^ with a large increase among black women.^61^ Among HDPs, pre-eclampsia has been referred to as “the great obstetrical syndrome,”^62–64^ with plausible mechanisms including intravascular inflammation, endothelial cell activation, and syncytiotrophoblast stress.^65^ Imbalances in circulating angiogenic factors which regulate angiogenesis and vessel remodeling play a pathogenic role with increased circulating soluble fms-like tyrosine kinase 1, which binds placental growth factor and vascular endothelial growth factor. This triggers the onset of pre-eclampsia by inducing microangiopathy.^64–66^ Pre-eclampsia and CVD share the same risk factors including diabetes, hypertension, and obesity with a 30% risk of chronic hypertension in the decade after birth.^64^ The unifying theme touches on a multitude of organ systems, with significant lifelong sequalae for the fetus and the pregnant person.^67–69^ The risk of complications has been estimated to be double for CVD and five times higher for the development of hypertension. The greatest risk is observed in those with recurrent preeclampsia in subsequent pregnancies or those with early-onset pre-eclampsia requiring delivery before 34 weeks’ gestation.^70–72^ Establishment of long-term follow-up is encouraged to not only continue to monitor BPs but also assess and treat modifiable cardiometabolic risk factors.

In 2019, updates on the primary prevention of CVD guideline indicated that a history of HDPs was a risk-enhancing factor requiring statin therapy for atherosclerotic CVD prevention.^73^ As mentioned earlier, the American College of Cardiology/American Heart Association guideline lowered the stage 1 hypertension threshold from 140/90 mm Hg to 130/80 mm Hg.^73^ As of 2022, the Society for Maternal-Fetal Medicine has revised the recommendation for treatment of chronic hypertension in pregnancy to lower BP to a target goal of less than 140/90 mm Hg.^74^ The National Institute for Health and Care Excellence guideline recommends treatment for greater than or equal to 140/90 mm Hg with a goal of less than 135/85 mm Hg.^75^ The International Society for the Study of Hypertension in Pregnancy recommends treatment to maintain a goal of 110–140/80–85 mm Hg.^76^ Based on available data, BP management in pregnancy indicates the benefits of lower treatment thresholds outside of pregnancy.

### Management of Categories of HDP

The categories of HDPs, defined by the American College of Obstetricians and Gynecologists Hypertension in Pregnancy Task Force diagnostic criteria and management are summarized in Table 2.

## SELECTION OF ANTIHYPERTENSIVE AGENTS IN PREGNANCY

Specific antihypertensive therapy is required for: (1) chronic treatment to gradually lower BP to maintain goal range, or (2) acute lowering of severe-range BP. The timing and setting of intervention along with the selection of antihypertensive agents will differ between these scenarios. The most used antihypertensive agents are listed in the Supplemental Table (available online at http://www.mayoclinicproceedings.org). Labetalol or nifedipine extended release are preferred for the long-term treatment of hypertension in pregnancy.^55^ Methyldopa, previously considered first-line, has shown lower efficacy with greater adverse effects.^77^ Diuretics can be considered second- or third- line HDP treatment, and their use during pregnancy and breastfeeding is generally tolerated but should be closely monitored.^78^ Angiotensin-converting enzyme (ACE) inhibitors and angiotensin receptor blockers are avoided due to potential teratogenic risks and associations with fetal growth restriction and neonatal kidney failure.^79^ There is evidence demonstrating the risk of beta-blockers, primarily atenolol, and its association with small for gestational age infants.^79^

For severe hypertension (systolic ≥160 mm Hg or diastolic ≥110 mm Hg) and if it is persistent (>15 minutes) requires prompt pharmacologic treatment. Therapy should be initiated within 30 minutes of diagnosis to decrease maternal risks. Medications used include intravenous labetalol, hydralazine, or oral immediate-release nifedipine.^80^ Hypertension disorders of pregnancy require timely treatment because CVA and hypertensive encephalopathy occur at lower BPs compared to outside of pregnancy. Pregnancies complicated by HDP should undergo increased antenatal surveillance for maternal health and risks to the neonate, including fetal growth restriction. Delivery timing depends on the underlying HDP diagnosis and the control of the disease process. The delivery mode (ie, vaginal vs cesarean birth) is not dictated by HDPs and is based solely on routine obstetrical indications.^52^

Postpartum Care for HDP

Blood pressure commonly decreases immediately after birth and declines for a few days postpartum but, after 3 to 5 days, there is a gradual increase in BP that often reaches levels higher than those observed during the antepartum period. The etiology is thought to be due to the mobilization of extravascular fluid leading to an increase in intravascular volume. Because the postpartum period is high-risk for pregnancies with HDPs, an outpatient follow-up within 1 to 2 weeks is recommended.^81^

Antihypertensive medications are titrated more liberally postpartum, with goals similar to those for nonpregnant woman. The majority of antihypertensive medications can be used safely during breastfeeding, with very low concentrations secreted in breastmilk.^82^ Angiotensin-converting enzyme inhibitors, including enalapril and captopril in low doses, have also shown low concentrations in breastmilk and can be considered an additional second- or third-line therapy if necessary as long as higher doses are avoided.^82^ Achieving optimal BP targets over the entire life cycle and establishing long-term follow-up are encouraged to not only continue to monitor BP but also to assess and treat modifiable cardiorenal metabolic risk factors and HMOD.^55,56^ The recommendation to work concomitantly with lactation consultants and maternal-fetal medicine physicians for specific medication and individualized care to the postpartum patient is imperative (Table 2).^55,56^

## HYPERTENSION AND MENOPAUSE

Changes in BP after menopause seem to be related to alterations in estrogen and progesterone levels along with other factors including genetic predisposition, obesity, type 2 DM, endothelial dysfunction, salt sensitivity, and arterial stiffness.^83^

Estrogen plays a significant role in the observed sex differences in hypertension and CVD.^16,84^ The mechanism for its vascular protective role is complex, and many pathways remain to be clarified. Estrogen contributes to vascular homeostasis by upregulation of endothelial nitric oxide pathway, augmenting prostacyclin release, reducing oxidative stress and fibrosis, and stimulating angiogenesis. Decline in estrogen has been associated with augmentation of ACE and angiotensin II pathways and lower MR expression.^32,85^ Estradiol deficiency results in RAAS system dysregulation with switch to proinflammatory pathways that contributes to impaired immune response and CVD.^86^ Estrogen modulates sympathetic tone by attenuating α-adrenergic receptor-mediated vasoconstriction, while enhancingβ-adrenergic receptor effect. This may be a cause of age-related increase in hypertension among women that is at least in part caused by falling estrogen levels during menopause.^16,84^

Although observational studies in humans and experimental studies in animals provide evidence that estrogen replacement protects post-menopausal women against CVD, randomized control trials did not support this concept.^87^ The reason for the disparity remains unclear; yet, it is hypothesized that several factors, including the type of estrogen used, interaction with progesterone, women’s age, and timing of the treatment, contribute to these results.^88,89^ There is evidence that estrogen therapy may be cardioprotective if started around menopause transition and may be harmful if started >10 years after menopause.^90^ Replacement with endogenous 17 β-estradiol shows superior cardiovascular benefit compared to replacement with oral conjugated equine estrogen, which is associated with a greater risk of hypertension development.^91,92^ Data on possible benefits of hormone replacement therapy on CVD are controversial.^93–95^

International medical societies, including the American Association of Clinical Endocrinologists, Endocrine Society, American College of Obstetricians and Gynecologists, and North American Menopause Society recommend against menopausal hormone therapy for primary or secondary prevention of CVD, especially for those older than 60 years or more than 10 years after menopause.^96^ Estrogen is a safe option for treatment of menopausal symptoms when initiated in healthy women younger than 60 years of age or within 10 years of menopause onset when it is used at the lowest effective dose and for the shortest total duration based on risk benefit analysis (typically <5–10 years).^97^

However, estrogen may have a plaque-destabilizing effect in the setting of advanced atherosclerosis, favoring thrombosis. Therefore, it is contraindicated in women with atherosclerotic CVD, BP greater than 180/110 mm Hg, venous thrombosis or pulmonary embolism, cerebrovascular disease, and congenital heart disease.^98^ Selected younger (<60 years of age) women with one or more cardiovascular risk factor including obesity, controlled hypertension, diabetes, and dyslipidemia may use menopausal hormone therapy; however, the transdermal route of estrogen is preferred and there should be an emphasis on optimizing primary prevention efforts. Additionally, cardiovascular risk must be regularly reassessed.^93–95^ Additional studies are needed to better understand the consequences of menopause on CVD and to determine the value, timing, and dosing of hormone replacement therapy on BP.

## HYPERTENSION IN TRANSGENDER INDIVIDUALS

Transgender patients receiving gender-affirming hormonal therapy may suffer from higher rates of CVD than cisgender people.^99^ Transgender women are generally prescribed a combination of estrogen and an antiandrogen if they have not had orchiectomy, whereas transgender men are generally only on testosterone. In transgender women, the choice of antiandrogen has a profound effect on BP.^100^ For example, decreased BP has been noted with spironolactone and increased BP has been observed with cyproterone acetate.^100,101^ In transgender men, BP increases after initiation of testosterone therapy; however, the incidence of hypertension in younger transgender men is low and comparable to the general population.^100,101^ One study found that transgender men receiving long-term testosterone exhibit higher aging-related arterial stiffness compared to cisgender women and cisgender men, suggesting a potential deleterious effect of testosterone on arterial function.^102^ The effect of male sex hormones in arterial vessels may be direct or indirect through estrogen originating from the peripheral aromatization of testosterone. Prospective studies evaluating the long-term effects of gender-affirming therapy on BP and HMOD are needed.

## RACIAL AND ETHNIC DIFFERENCES IN WOMEN WITH HYPERTENSION

The prevalence of hypertension in the United States may vary by race and ethnicity. Specifically, the prevalence of hypertension in Black individuals is higher (45.3%) compared to either White individuals (31.4%, *P*<.001), Hispanic individuals (31.6%), or Asian American individuals (31.8%).^103^ The discrepancy may partly be due to differences in lifestyle, genetic influences, and economic status. In addition, differences in prevalent rates may be an underlying contributor to the higher hypertension-related complications in Black persons that include CKD, CVD, stroke, heart failure, and death compared to other racial and ethnic minority groups.^103,104^

Furthermore, the prevalence of hypertension in Black women is higher than in Black men. Recent studies reported the prevalence of hypertension among Hispanic women as 14.0% and 39.0% among non-Hispanic Black women.^105^ Looking at trends in hypertension incidence solely among women, Hispanic women present with the second highest incidence of hypertension after non-Hispanic Black women.^106^

Rates of hypertension awareness tend to be higher in White compared to Black patients, but hypertension control rates are lower across all racial and ethnic groups, possibly due to systemic racial discrimination, differences in access to health care, socioeconomic factors, and lack of trust in the health care system.^103,104^ Although Black women have higher hypertension prevalence, they have similar rates of hypertension awareness and control compared to men. Among all racial and ethnic minority women, Hispanic women are reported to achieve the highest hypertension control (61.7%) with medications, followed closely by Non-Hispanic White (59.6%) and Non-Hispanic Black women (41.5%).^105^

In Black women, depression is associated with both hypertension and the lack of healthy lifestyle promoting behaviors.^107^ Psychosocial factors, such as lower income level, greater number of comorbidities, lower coping mechanisms and resilience, and poorer medication adherence were significantly associated with higher depression scores, contributing to higher levels of hypertension.^107,108^ Psychological stressors, including discrimination, financial stress, and reduced caregiving are further associated with higher rates of hypertension in Black women.^108^ Social determinants of health such as health literacy, patient activation, and financial stress are more prevalent barriers to optimal BP control, particularly in Black women compared to their White counterparts.^109^

Future strategies to achieve better control of hypertension among racial and ethnic minority women may begin with appropriate and culturally sensitive screening approaches, increasing awareness, and improving access to appropriate use of antihypertensive medication. Importantly, approaches to achieve more equitable management may include identification of barriers to care, particularly among women of reproductive age.

## LIFESTYLE MODIFICATIONS AND HYPERTENSION MANAGEMENT IN WOMEN

Lifestyle modification plays a pivotal role in preventing and managing hypertension. In fact, 24.3 million (21%) adults with hypertension can be treated with lifestyle modifications alone, without the need for antihypertensive medications.^110^

### Nutrition and Hypertension Management in Women

The Dietary Approaches to Stop Hypertension (DASH) diet has emerged as a balanced dietary strategy for preventing and treating hypertension.^111^ This diet emphasizes fruits, vegetables, and low-fat dairy products and recommends reduced amounts of saturated fat, total fat, and cholesterol and is rich in potassium, magnesium, calcium, and fiber. Importantly, the DASH diet lowers BP beyond the level achievable by simply reducing sodium intake. It has been demonstrated that it not only results in the expected lower BP, but can benefit cardiometabolic health, decreasing body mass index (BMI), body fat content, fasting glucose, insulin, and leptin concentrations.^112,113^

The consensus from studies on sex differences in dietary patterns and hypertension outcomes remain unclear. One study on sex differences in those between 40 and 69 years of age found that diets higher in the consumption of vegetables, potatoes, fruits, beans, and seaweeds were related to lower BP.^114^ Another study concluded a diet rich in whole grains and legumes was inversely associated with the risk of hypertension in women, suggesting sex differences in association with diet and hypertension.^115^ Additional studies are needed to evaluate diet-based recommendations for women in the various stages of life cycle relative to hormonal alterations, aging, sodium retention after menopause and by race and ethnicity.

Impact of Alcohol on Hypertension Management in Women

The effect of chronic alcohol intake on BP in women is understudied. The relationship between alcohol intake and BP is complex and might vary at different stages of a woman’s life cycle. In healthy premenopausal women 25 to 49 years of age, a randomized controlled intervention study confirmed dose-dependent effects of alcohol on ambulatory BP.^116^ These findings verified that regular consumption of alcohol (200–300 mL red wine/d, 146–218 g alcohol/wk) can elevate 24-hour systolic and diastolic BPs. This confirmed that the magnitude of the increase in BP is similar to that previously reported in men.^116^ Although drinking within “sensible limits” of 1 to 2 drinks/d seems to have no negative impact on chronic hypertension,^117^ additional studies to investigate the impact of alcohol on women’s BP and arterial stiffness in different stages of the life cycle are needed.

### Impact of Cigarette Smoking on Hypertension in Women

Smoking remains the single most important preventable cause of cardiovascular morbidity and mortality worldwide. Earlier data in a large cohort of women smokers suggested a modest association between developing hypertension and smoking, with the strongest effect among women who smoke at least 15 cigarettes per day.^118^ Smoking seems to be more harmful in women than men, particularly women who use oral contraceptives.^119^ Results from a European study on life expectancy suggest that women who smoke, like men, have a relatively higher risk of reduced life expectancy.^120^ There is a need for sex-based studies to address the gaps in knowledge and determine whether these differences are rooted in biological factors, linked to variations in smoking behavior, or influenced by factors such as BMI, age, and hormonal status.

### Weight and Body Mass and Hypertension in Women

Increased BMI greater than 25 kg/m^2^ is associated with greater risk of hypertension and mortality, whereas weight loss can reduce risk of hypertension.^121,122^ Data support up to a 20-mm Hg drop in BP with 10 kg of weight loss.^123^ Combined use of DASH diet and weight loss can further lower BP.^5^ Results from a study on women who had a BMI greater than 25 kg/m^2^ at the age of 18 years showed a relative risk of 2.2 for developing hypertension.^121^ Recent data indicate that indices of central adiposity including waist circumference (WC), and waist-to-height ratio (WHtR), may have better predictive value for the risk of hypertension in women.^124^ The WC and waist to hip ratio and WHrR may increase with parity and is consistently associated with an increase in WC and a reduction in hip circumference in women 18 to 30 years of age.^125^

### Impact of Physical Activity on Hypertension in Women

Approximately 80% of US adults are insufficiently active. Regular, mild-to-moderate aerobic activity can independently decrease BP by 5 to 8 mm Hg in women, regardless of weight loss.^126,127^ Few studies have assessed the combined impact of body weight and physical activity in relation to hypertension among women. A report studying a large cohort of French women concluded that higher physical activity was associated with a lower risk of hypertension, but only within a BMI range of 22.5 to 25.0 kg/m^2^.^128^ Further studies are needed to provide guidance on the duration and intensity of physical activity on BP reduction and HMOD in women.

### Impact of Sleep on Hypertension in Women

**FIGURE 1.**
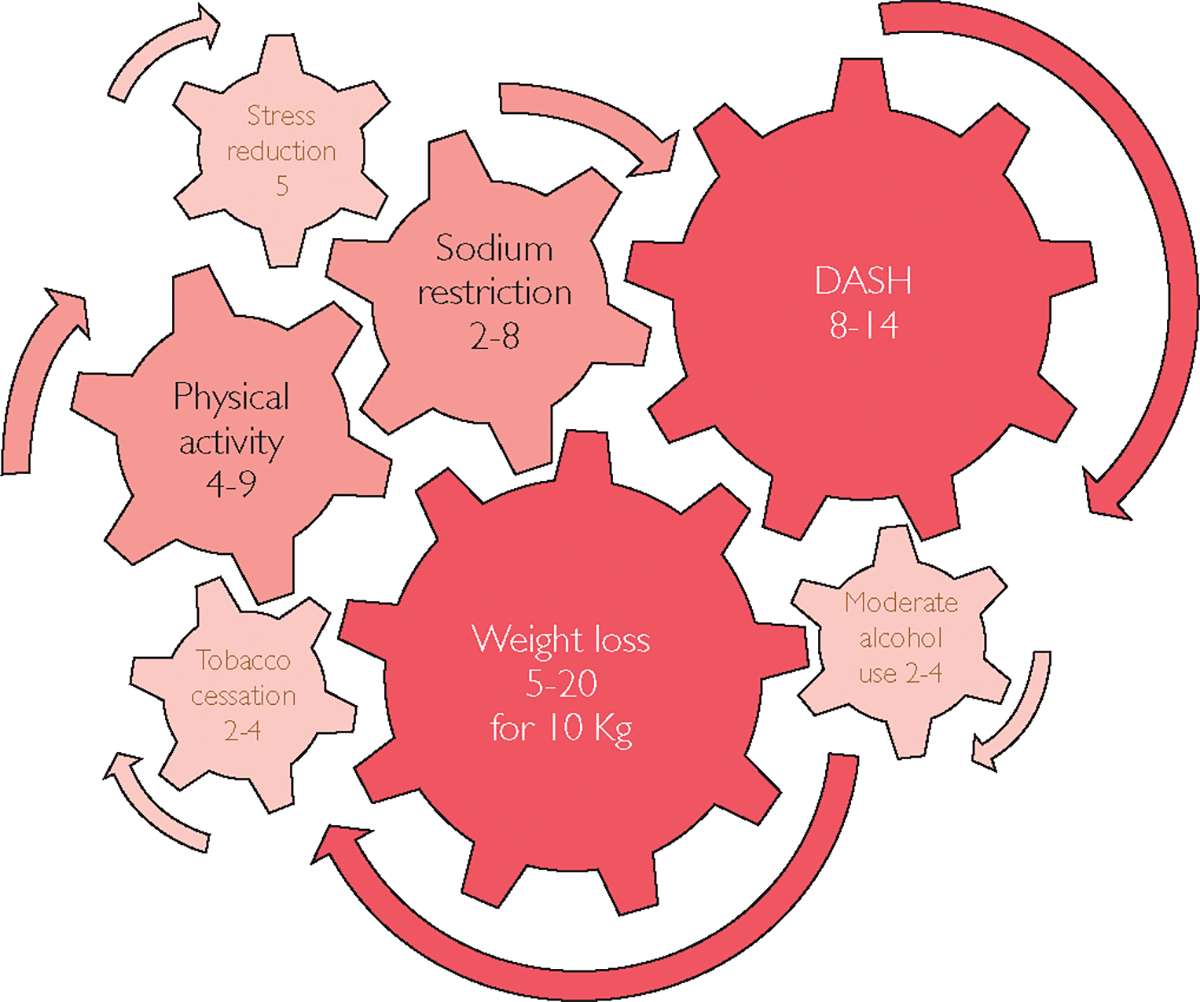
Lifestyle modifications have potential positive impacts that measurably reduce blood pressure. Data from the American College of Obstetricians and Gynecologists.^5^ DASH, Dietary Approaches to Stop Hypertension.

Sleep disturbances are associated with increased risk of morbidity and mortality.^129^ Key sleep disorders that can impact BP are obstructive sleep apnea (OSA), short sleep duration, and poor sleep quality.^130–132^ One study of 277 perimenopausal women with a mean age of 56 years and a mean BMI of 28 kg/m^2^ showed that women with moderate to severe OSA were more likely to be hypertensive, use more medications to reduce BP, and have higher awake and nocturnal BP and increased arterial stiffness.^133^ Results from the Nurses’ Health Study supported the need for sufficient sleep to reduce hypertension incidence and prevalence.^131^ The prevalence of hypertension was significantly higher among women who slept 5 hours or less per night. Studies suggest that night shift work, short sleep duration, or poor sleep with circadian disruption might increase the risk of hypertension because acute sleep restriction has been shown to increase BP and sympathetic nervous system activity.^131,134^ Figure 1 summarizes the effects of lifestyle modifications on BP with the largest impacts being from the DASH diet and weight loss.^5^

## PSYCHOLOGICAL PARAMETERS IN WOMEN WITH HYPERTENSION

The association between mental health and development of hypertension is complex.^135^ Presence of depression, anxiety, or chronic stress, and particularly severe mental illness is associated with hypertension and other cardiovascular risk factors.^136^ Psychosocial factors that induce emotional stress can evoke a physiological response meditated by activation of sympathetic nervous system, inflammation, and the hypothalamic–pituitary–adrenal axis.^137,138^ Factors such as hostility and job-related stress have been found to be associated with higher circulating catecholamines, higher cortisol, and increased BP over time.^139^

Comorbid depression, anxiety, and severe mental illness with hypertension share common risk factors and neurobiological mechanisms such as reduced baroreflex sensitivity due to autonomic dysfunction caused by chronic stress. Cardio- and cerebrovascular risk factors (eg, obesity, smoking, poor sleep, chronic stress, and poor lifestyle habits) are higher in those with mental illness, including depression and anxiety.^136,140–143^ A cohort study of women veterans showed that post-traumatic stress disorder was associated with increased risk of ischemic heart disease in these women.^144^ This may have implications for ischemic heart disease risk assessment in vulnerable individuals.

Menopause is the time of a woman’s life cycle recognized by biological and social changes which can impact mental wellbeing and risk of hypertension and CAD.^145^ Menopause transition has an additional adverse effect to aging that may demand specific attention to ensure optimal cardiovascular health and quality of life.^145^ Stress and depression management can play a complementary role to lifestyle modification to decrease BP.^146^ Studies suggest stress-reducing interventions, such as transcendental meditation, decrease diastolic and systolic BPs.^146,147^ Some potential confounders in former studies can be psychosocial determinants of health such as socioeconomic status, access to health care, obesity, history of chronic stress, and trauma.^140,141^ To address race-related stressors, such as racial discrimination, a review from 2015 highlights promising interventions that can reduce the effects of discrimination on health, such as religious involvement, value affirmation, forgiveness, and racism counter marketing.^148^

## PHARMACOTHERAPY TREATMENT FOR WOMEN WITH HYPERTENSION

**FIGURE 2.**
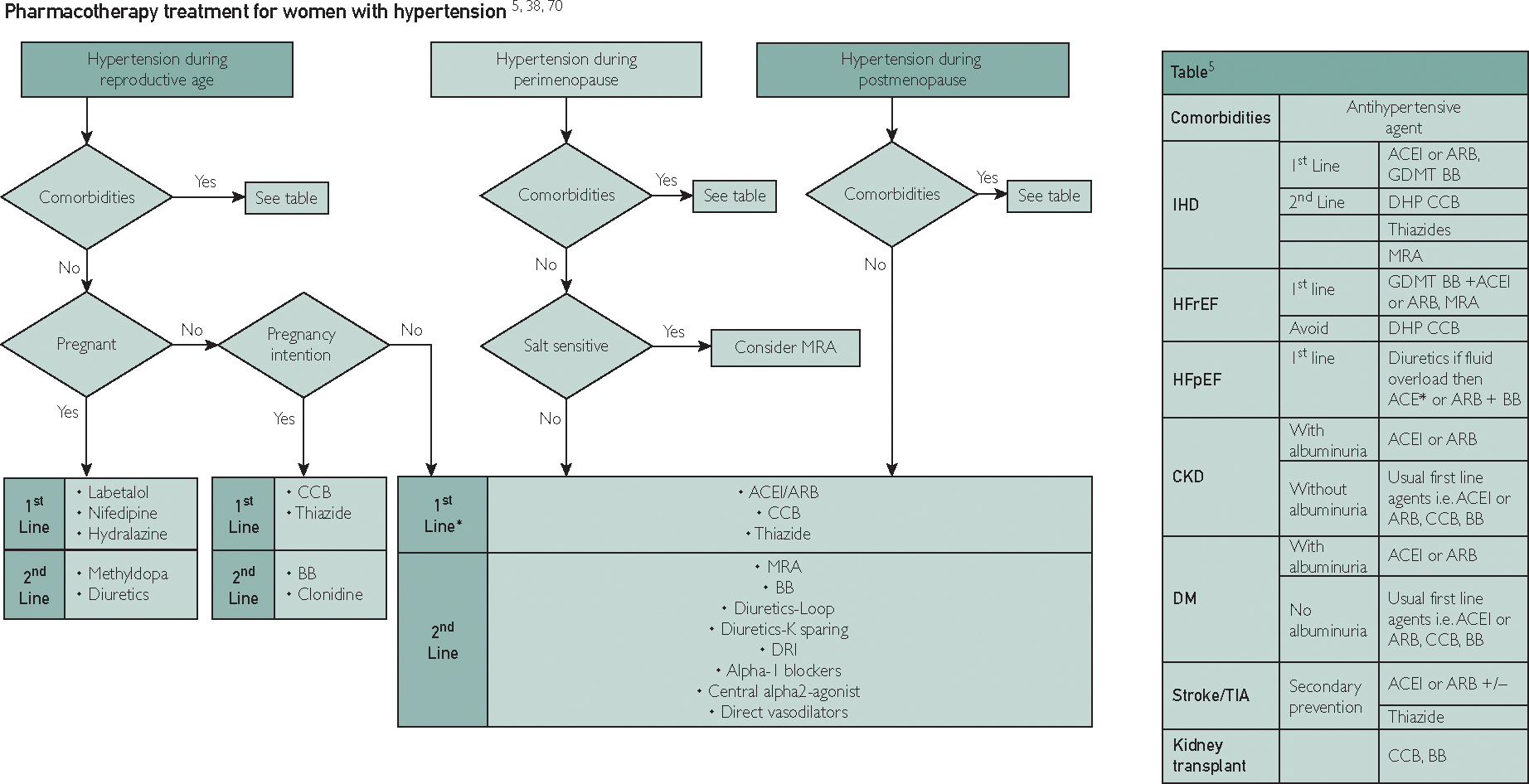
Treatment algorithm for antihypertensive medication in women during three phases: reproductive, perimenopause, and post-menopause and relation to comorbidities.^5,32,55,79^ ACE, angiotensin-converting enzyme; ACEI, angiotensin-converting enzyme inhibitor; ARB, angiotensin receptor blocker; BB, beta-blocker; CCB, calcium channel blocker; DHP, dihydropyridine; GDMT, guideline-directed medical therapy.

Antihypertensive therapy choices vary among women across different phases of life. Therapy choices are limited during the reproductive age including the preconception phase and during pregnancy given the risk of teratogenicity and the side effects on the fetus. This limits access to RAAS blockade agents for patients with diabetes, CKD, or cardiac disease.^55,79^ Therapy choices during perimenopause and post menopause are more similar to those with men in relation to comorbidities; there is greater attention to MR antagonists as a preferential treatment for premenopausal women who have been diagnosed with salt-sensitive hypertension with recent evidence that aldosterone production is sex-specifically heightened in salt-sensitive hypertensive women.^5,32^ Figure 2 summarizes pharmacotherapy treatment for women across three phases of life.^5,32,55,79^ Adverse effects of antihypertensive therapy can be higher in women than men with some class of medications such as ACE inhibitor–induced cough or edema with calcium antagonists. Table 3 summarizes sex-specific adverse events of antihypertensive agents.^5,149,150^

## CONCLUSION

There is a significant clinical need for the diagnosis and optimal management of hypertension in women across the lifespan. With the continued growth of hypertension in women and the anticipated increase in prevalence of HDPs due to the obesity epidemic, increasing maternal age, and the rising prevalence of metabolic syndrome, additional strategies in a multidisciplinary fashion are needed to address unmet needs.

## Supplementary Material

Supplemental Table

## Figures and Tables

**TABLE 1.** Secondary Causes of Hypertension and Gender Difference^a^

Cause	When to suspect	Laboratory screening	Sex difference

Primary aldosteronism^35^	Hypokalemia (28% of cases) Resistant hypertension Age <50 y.	Plasma renin activity (preferred if on estrogen) or plasma renin concentration and plasma aldosterone.	None
Renovascular disease^33,34^ Fibromuscular dysplasia 10% -30% Atherosclerotic renal artery stenosis 60%-90%	Severe hypertension in young and middle-aged women. It may also occur in men and children with resistant hypertension. Common in patients with atherosclerosis — higher in men older than 45 y.	Renal artery duplex ultrasound and/or CT angiography.	9-fold higher prevalence in women.
Drug-induced hypertension^36^	Sympathetic agent NSAIDs Corticosteroids CNS-stimulants Estrogen and progestins Contraceptives Dietary supplements (ginseng, licorice, yohimbine) SNRIS immunosuppressants		Dietary supplement and NSAIDs use are more common among women.
OSA^37,38^	Snoring Gasping Apneas Morning headache Fatigue Depression Women, tend to present in an atypical fashion with less frequent reports of snoring or witnessed apneas.	Polysomnography Home sleep apnea test	OSA is more common in men than women and the prevalence increases throughout life in both sexes.
Endogenous Cushing syndrome^29^	Higher degree of suspicion: wide purple striae, thin skin with easy bruising, proximal limb atrophy, and facial plethora. Common yet less specific characteristics: weight gain, excessive abdominal adiposity, fatigue, acne, hirsutism, and menstrual irregularities.	Overnight 1 mg dexamethasone suppression test cortisol 24-h urine free cortisol. Late-night salivary cortisol.	3-fold higher incidence in women.
Pheochromocytomas-paragangliomas^39^	Adrenal mass Familial genetic syndrome Classic symptoms of headaches, palpitations, pallor, and diaphoresis Nonischemic and nonvalvular cardiomyopathy, including Takotsubo cardiomyopathy Sustained or paroxysmal hypertension Normal BP in 5% to 15% of patients	Plasma free or urinary fractionated metanephrines	None
Severe thyroid dysfunction^40^	Hyperthyroidism: tachycardia, fine tremor, weight loss, increased bowel movements. Hypothyroidism: bradycardia, weight gain, new onset constipation, fatigue.	TSH, Free T4, and Free T3	10-fold higher prevalence in women
Severe primary hyperparathyroidism^41^	Hypercalcemia Nephrolithiasis Low bone mineral density Neuropsychologic symptoms	PTH and calcium levels	2–3-fold higher incidence and prevalence in women
Acromegaly^42^	Enlargement of acral tissues, frontal bossing, and mandibular prognathism Increased sweating Headache Worsening or new onset type 2 diabetes mellitus Obstructive sleep apnea	Screening IGF-1 level Confirmatory: growth hormone suppression test to an oral glucose load	None

a BP, blood pressure; CNS, central nervous system; CT, computed tomography; IGF, insulin-like growth factor; NSAIDs, nonsteroidal anti-inflammatory drug; OSA, obstructive sleep apnea; PTH, parathyroid hormone; SNRIS, serotonin and norepinephrine reuptake inhibitor; TSH, thyroid stimulating hormone.

**TABLE 2.** Hypertensive Disorders of Pregnancy^a,b^

Condition	Diagnostic criteria	Management

Chronic hypertension	BP: systolic BP ≥140 mm Hg or diastolic BP ≥90 mm Hg on two occasions at least 4 h apart any time before 20 wks of pregnancy, including outside of pregnancy	Well-controlled, no medication, expectant management 38 0/7– 39 6/7-wks' gestation Well-controlled, with medication, expectant management 37 0/7– 39 6/7-wks' gestation Difficult to control, medication, expectant management 36 0/7– 37 6/7-wks' gestation
Gestational hypertension	BP: systolic BP ≥140 mm Hg or diastolic BP ≥90 mm Hg on two occasions at least 4 h apart after 20 wks with previously normal BPs	Expectant management up to 37 0/7 wks' gestation or at diagnosis, if later
Preeclampsia without severe features	BP: systolic BP ≥140 mm Hg or diastolic BP ≥90 mm Hg on two occasions at least 4 h apart after 20 wks with previously normal BPs and Proteinuria: ≥300 mg/24-hour urine collection or protein/creatinine ratio ≥0.3 or dipstick reading 2+, if quantitative methods are not available	Expectant management up to 37 0/7 wks' gestation or at diagnosis if later
Pre-eclampsia with severe features	BP: systolic BP ≥160 mm Hg or diastolic BP ≥110 mm Hg on two occasions at least 4 h apart after 20 wks with previously normal BPs or Systolic BP ≥140 mm Hg or diastolic BP ≥90 mm Hg on two occasions at least 4 h apart after 20 wks with previously normal BPs with evidence of end-organ damage, including: Thrombocytopenia (platelet <100 x 10^9^/L), Renal insufficiency (creatinine >1.1 mg/dL or doubling in serum creatinine in the absence of other renal disease) Impaired liver function (transaminases twice normal), or severe persistent epigastric pain unresponsive to pain Pulmonary edema New-onset headache unresponsive to medication Visual disturbances	Expectant management up to 34 0/7 wks or at diagnosis if later unless any contraindication to expectant management, including eclampsia, fetal demise, non-reassuring fetal heart tracing, placental abruption, disseminated intravascular coagulation, renal insufficiency, HELLP syndrome, or persistent symptoms of severe pre-eclampsia
HELLP syndrome	May include elevated BPs: systolic BP ≥140 mm Hg or diastolic BP ≥90 mm Hg. Lactate dehydrogenase ≥600 IU/L Transaminases twice normal Platelets count <100 x 10^9^/L	Maternal stabilization Delivery at diagnosis soon after maternal stabilization

a BP, blood pressure; HELLP, hemolysis, elevated liver enzymes, low platelet count.

b Elevated Transaminases: Aspartate aminotransferase (AST) or alanine aminotransferase (ALT) levels ≥70 U/L, or at least twice the upper limit of normal.

From the American College of Obstetrics and Gynecology.^55,56^

**TABLE 3.** Sex-Specific Adverse Events of Antihypertensive Agents

Antihypertensive agent	Sex-specific adverse events	Additional benefit

Angiotensin-converting enzyme inhibitor	Potential risk in women in childbearing ages due to risk of fetal abnormalities Three-fold higher cough	Suitable in patients with comorbidities such as chronic kidney disease, coronary artery disease, congestive heart failure, diabetes mellitus, proteinuria
Angiotensin receptor blocker	Potential risk in women in childbearing ages due to risk of fetal abnormalities	Migraine prevention
Dihydropyridine calcium channel blocker	More peripheral edema in younger women	Suitable choice for Raynaud
Non-dihydropyridine calcium channel blocker	More peripheral edema in younger women	Verapamil for prevention of migraine Might improve proteinuria
Thiazide diuretics	Hyponatremia and hypokalemia more common in women Decreases placental perfusion, and plasma volume and association with pre-eclampsia	Reduce risk of osteoporosis Beneficial in calcium containing kidney stones
Mineralocorticoid receptor antagonist	Feminization of male fetus Oligohydramnios Failure of kidney development Ambiguous genitalia in newborns	Resistant hypertension Suitable in patients with comorbidities such as primary hyperaldosteronism, congestive heart failure, potassium wasting tubulopathies. Beneficial in androgen dependent conditions such as acne, hirsutism, polycystic ovarian syndrome Spironolactone reduces testosterone and is useful in male-to-female transgender
β-blockers	Sexual dysfunction	Migraine prevention Beneficial in patients with comorbidities such as ischemic heart diseases, coronary artery disease, congestive heart failure, atrial fibrillation
Minoxidil	Hirsutism	Resistant hypertension

From Whelton et al^5^, Bager et al^149^, and Ahmad et al.^150^

## ARTICLE HIGHLIGHTS

- Hypertension and its sequalae in women should be managed by a multidisciplinary team based on the phase of a woman’s life cycle, underlying medical comorbidities, socioeconomical stressors, and access to care.
- Choices of therapy should be made in relation to reproductive, peri-, and post-menopausal phases, with consideration of hormonal alterations and other comorbid factors such as hypertension disorders of pregnancy.
- Researchers are encouraged to incorporate stratification by sex, race, ethnicity, and the phase of the woman’s life cycle when designing and analyzing clinical trials focused on dietary patterns, lifestyle modifications, and the efficacy of pharmacotherapy.
- Several challenges and unmet needs to optimize the management of hypertension in women still exist.

## Abbreviations and Acronyms:

ABPM: ambulatory blood pressure monitor
ACC: American College of Cardiology
AHA: American Heart Association
BMI: body mass index
BP: blood pressure
CAD: coronary artery disease
CKD: chronic kidney disease
CVD: cardiovascular disease
DASH: Dietary Approaches to Stop Hypertension
HDP: hypertension disorders of pregnancy
HMOD: hypertension-mediated organ damage
MR: mineralocorticoid receptor
OSA: obstructive sleep apnea
PA: primary aldosteronism
PRA: plasma renin activity
PRC: plasma renin concentration
RAAS: renin-angiotensin-aldosterone system
WC: waist circumference
WHtR: waist-to-height ratio
