# Supplementary material for: Hypertension Management in Women With a Multidisciplinary Approach
Source: Mayo Clin Proc. Author manuscript; Available in PMC 2025 Apr 22. (PMC12013344; doi:10.1016/j.mayocp.2024.10.005)
Supplement: Supplemental Table [file NIHMS2066954-supplement-Supplemental_Table.pdf]

Supplemental Table. Anti-hypertensive Agents in Pregnancy.[1,2]

Drug	Class	Dosing	Comments
**First-line**			
Labetalol	Combined α-adrenergic and β-adrenergic blocker	200-2400 mg/d by mouth in divided doses every 8-12 hours<br><br>Initial dose: 100-200 mg every 12 hours	Avoid in patients with moderate-to-severe asthma, decompensated heart failure, high grade AV block, or significant bradycardia/sinus node dysfunction
Nifedipine extended-release	Calcium channel blocker	30-120 mg/d by mouth<br><br>Initial dose: 30-60 mg once daily	Do not use sublingual form<br><br>Avoid in patients with tachycardia
Hydralazine	Vasodilator	40-300 mg/d in divided doses every 6-8 hours<br><br>Initial dose: 10 mg every 6 hours	Can causes reflex tachycardia and fluid retention
**Second- or third line**			
Methyldopa	α-adrenergic agonist	500-3,000 mg/d by mouth in divided doses every 6-12 hours<br><br>Initial dose: 250 mg every 8-12 hours	May not be as effective as other antihypertensive medications.<br><br>Use limited by side effect profile (sedation, depression, dizziness)
Hydrochlorothiazide	Diuretic	12.5-50 mg/d by mouth<br><br>Initial dose: 12.5-25 mg once daily	May cause intravascular volume depletion. Monitor electrolytes.
Clonidine	α-adrenergic agonist	0.2-0.6 mg in divided doses every 8-12 hours<br><br>Transdermal: 0.1-0.3 mg every 7 days	May cause rebound hypertension<br><br>Transdermal patch can be used in patient who cannot take oral antihypertensive drugs

References:

1. American College of O, Gynecologists' Committee on Practice B-O. ACOG Practice Bulletin No. 203: Chronic Hypertension in Pregnancy. Obstet Gynecol. Jan 2019;133(1):e26-e50. doi:10.1097/AOG.0000000000003020

2. . Gestational Hypertension and Preeclampsia: ACOG Practice Bulletin, Number 222. Obstet Gynecol. Jun 2020;135(6):e237-e260. doi:10.1097/AOG.0000000000003891
